# Enhanced Imaging in Scanning Transmission X-Ray Microscopy Assisted by Ptychography

**DOI:** 10.3390/nano15070496

**Published:** 2025-03-26

**Authors:** Shuhan Wu, Zijian Xu, Ruoru Li, Sheng Chen, Yingling Zhang, Xiangzhi Zhang, Zhenhua Chen, Renzhong Tai

**Affiliations:** 1 Shanghai Institute of Applied Physics, Chinese Academy of Sciences, Shanghai 201800, China; wushuhan@sinap.ac.cn (S.W.);; 2 Shanghai Synchrotron Radiation Facility, Shanghai Advanced Research Institute, Chinese Academy of Sciences, Shanghai 201204, China; 3 University of Chinese Academy of Sciences, Beijing 100049, China; 4 School of Physical Science and Technology, Shanghai Tech University, Shanghai 201210, China

**Keywords:** STXM, ptychography, image enhancement, accurate probe reconstruction, deconvolution

## Abstract

Scanning transmission X-ray microscopy (STXM) is a direct imaging technique with nanoscale resolution. But its resolution is limited by the spot size on the sample, i.e., by the manufacturing technique of the focusing element. As an emerging high-resolution X-ray imaging technique, ptychography utilizes highly redundant data from overlapping scans as well as phase retrieval algorithms to simultaneously reconstruct a high-resolution sample image and a probe function. In this study, we designed an accurate reconstruction strategy to obtain the probe spot with the vibration effects being eliminated, and developed an image enhancement technique for STXM by combining the reconstructed probe with the deconvolution algorithm. This approach significantly improves the resolution of STXM imaging and can break the limitation of the focal spot on STXM resolution when the scanning step size is near or below the spot size, while the data processing time is much shorter than that of ptychography. Both simulations and experiments show that this approach can be applied to STXM data at different energies and different scan steps using the same focal spot retrieved via ptychography.

## 1. Introduction

Scanning transmission X-ray microscopy is a synchrotron-based X-ray imaging technique that combines high-resolution spectroscopy with nano-imaging as a powerful means of characterization [1,2,3,4,5,6]. The world’s first STXM system was built by Horowitz and Howell at the Cambridge Electron Accelerator in 1972 [7]. STXM samples can be in the form of solids, liquids, or particles on the micron scale in materials, life, energy, and environmental sciences. In this technique, incident X-rays are focused by a Fresnel zone plate (FZP) to create a nano-sized probe spot that is irradiated onto the sample for point-by-point scanning. A downstream detector records the transmitted light intensity point-by-point, which is organized by a computer to form a transmission image of the sample. These images provide detailed chemical and structural information of the sample with high spatial resolution [8,9]. The STXM resolution is determined by a combination of the FZP focusing capability and the mechanical scanning accuracy, but ultimately depends on the focal spot size, typically down to 30 nm. The STXM can also be combined with a variety of other signals, such as total electron yield and fluorescence, among others, to provide extended imaging capabilities [10,11].

In 1999, Miao et al. achieved the first X-ray coherent diffraction imaging (CDI) of non-periodically aligned gold nanoparticles using a high-brightness, highly coherent synchrotron radiation source [12]. As a high-resolution lensless imaging technique, CDI has undergone rapid development over the past two decades. It is an extension of the traditional X-ray crystallography method for non-crystalline samples [13]. In this method, the sample is irradiated with a beam of coherent light, the resulting diffraction pattern is collected with oversampling in the far field, and the real-space sample image is reconstructed from the diffraction pattern by an iterative phase retrieval algorithm. Unlike STXM, the resolution of CDI is not limited by the focusing element, but by the X-ray wavelength and the maximum detectable diffraction angle, thus giving CDI a much higher resolution than STXM. As a scanning version of CDI, ptychography was first proposed by Rodenburg and Faulkner in 2004 [14,15]. Due to its ability to image extended samples and its fast convergence of reconstruction, ptychography has gradually become a widely used branch of CDI [16,17]. In this method, due to the high overlap between the exposure positions of the incident light on the sample (usually the linear overlap is greater than 70%), the resulting diffraction patterns have high information redundancy, so that the high-resolution sample image and the high-precision illumination function can be reconstructed simultaneously from the dataset. Moreover, the redundancy greatly accelerates the convergence of the phase retrieval calculation. Currently, the ptychography resolution has reached 5–10 nm [18,19], and it has numerous applications in biology, materials, medicine, environment, chemistry, and other fields [20,21,22,23,24,25,26].

In general, the advantage of STXM is its high imaging speed, but its resolution is limited by the focusing element process; whereas the advantage of ptychography is its high resolution, however, its image reconstruction is complicated and time-consuming. Therefore, we combined STXM with ptychography to develop STXM image enhancement technology. Currently, some relevant methods have been proposed for the deconvolution of the STXM images with modeled probes to improve the resolution [27,28,29], as well as the deconvolution of hard X-ray fluorescence images with a ptychography-reconstructed probe function [30,31].

In this work, we designed a reconstruction strategy for accurate probes and developed an STXM image enhancement technical scheme based on ptychography-reconstructed probes and the deconvolution method. This approach significantly improves the resolution of STXM, and can break the limitation of the focused spot size on the STXM resolution when the scanning step size is around the spot size. In the meantime, its data processing time is much shorter than that of ptychography. Both simulations and experiments show that this approach has resolution enhancement effects on STXM images with different scanning step sizes and at different energies.

## 2. Principle and Methodology

### 2.1. Fundamentals of Deconvolution

It is well known that the STXM is the scanning imaging on a regular grid, and the resulting image is actually the result of convolving the sample function with the probe function as follows:

(1) i(x,y) = o(x,y)∗p(x,y)

where $i\left(x,y\right)$ is the STXM image, $o\left(x,y\right)$ is the sample function, and $p\left(x,y\right)$ is the probe function. Using Equation (1) and the Fourier convolution theorem, the sample function can be derived from the STXM image and the probe function as follows:

(2) $$o\left(x,y\right)=\;F^{-1}\left\{\frac{I\left(f_{x},f_{y}\right)}{P\left(f_{x},f_{y}\right)}\right\}=F^{-1}\left\{\frac{F\left\{i(x,y)\right\}}{MTF(f_{x},f_{y})}\right\}$$

where $I\left(f_{x},f_{y}\right)=F\left\{i\left(x,y\right)\right\}$ represents the Fourier transform of the STXM image and $P\left(f_{x},f_{y}\right)$ is the Fourier transform of the intensity point spread function (the probe function), which is also the modulation transfer function, $MTF\left(f_{x},f_{y}\right)$. That is, the sample function can be recovered from the probe function and the STXM image via deconvolution. But deconvolution attempts to solve an ill-posed problem which is very sensitive to the additive noise in the image. For example, in Equation (2) the value of $P\left(f_{x},f_{y}\right)$ will become small in the high frequency range, which will cause the noise to be amplified, and result in a poor quality of the deconvolved image. A number of image deconvolution methods, such as the modified residual norm steepest descent algorithm [32], the Wiener filtering algorithm [27,33,34], the least squares method (LSQR) [35,36,37], and the Lucy–Richardson algorithm [38,39], have been proposed to overcome the above difficulty.

We mainly adopt the Wiener filtering algorithm in our deconvolution enhancement process. The basic equation is as follows [35]:

(3) $$o\left(x,y\right)=\;F^{-1}\left\{F\left\{i(x,y)\right\}D(f_{x},f_{y})\right\}$$

(4) $$D\left(f_{x},f_{y}\right)=\frac{1}{H(f_{x},f_{y})}\left[\frac{1}{1+S_{n}(f_{x},f_{y})/S_{f}(f_{x},f_{y})}\right]$$

where $D\left(f_{x},f_{y}\right)$ is the deconvolution filter, $H\left(f_{x},f_{y}\right)$ is the Fourier transform of the degradation transfer function (the point spread function), $S_{n}\left(f_{x},f_{y}\right)={\left|N\left(f_{x},f_{y}\right)\right|}^{2}$ is the power spectrum of the noise, and $S_{f}\left(f_{x},f_{y}\right)={\left|F\left(f_{x},f_{y}\right)\right|}^{2}$ is the power spectrum of the undegraded image. That is to say, by multiplying the Fourier transform of the STXM image with the deconvolution filter in the frequency domain and then performing the inverse Fourier transform, we can obtain the sample function. The variable parameters involved in this process are the noise-to-signal ratio $NSR=S_{n}/S_{f}$ and the point spread function (probe) $H\left(f_{x},f_{y}\right)$. Therefore, as long as we choose the correct probe function and the proper noise-to-signal ratio NSR, a high-resolution sample image can be obtained by deconvolving the STXM image with the probe.

In addition, this study also used the least squares method (LSQR) [36,37,38] and the Lucy–Richardson algorithm [39,40] for STXM deconvolution enhancement, and the results show that these methods are also effective in improving the resolution of the STXM images based on the ptychography-reconstructed probe.

### 2.2. Image Enhancement Strategy

#### 2.2.1. Accurate Reconstruction Strategy for Illumination Spot Based on Ptychography

The accurate point spread function (PSF) or the focal spot function is a key factor to precisely deconvolve the STXM intensity image. This PSF can be derived through ptychographic reconstruction of the probe wavefront. In this study, to obtain accurate probe functions, ptychography reconstructions were performed using the multi-probe-mode ePIE algorithm [40,41] coupled with a vibration separation method [42]. The basic idea of the multi-probe-mode ePIE is to decompose partially coherent light into multiple orthogonal modes which are mutually incoherent and combine these probe modes into the iterative phasing algorithm to obtain high-quality reconstructed images. The reconstructed probe function is a non-coherent superposition of the individual probe modes.

In this work, we designed an accurate ptychographic reconstruction strategy for probe wavefront to eliminate the effects of random vibration on ptychography reconstruction. The strategy begins with a multi-probe-mode reconstruction of the diffraction data to obtain an accurate sample function as well as a background noise function [43]. Here, six probe modes were used in the high-accuracy reconstructions. Then, the resulting sample function and the background function are input into the reconstruction algorithm as the initial sample function and the initial background function for the second reconstruction, which is a single-probe mode ePIE reconstruction. However, in this reconstruction, the vibration separation method [42] is introduced into the iterative algorithm, which segments the spatial distribution of the instantaneous vibration positions (IVPs) around each scanning position recorded by the laser interferometer. Each vibration position segment corresponds to a vibrational state of the sample, and the exit waves of all the sample vibrational states are incoherently superposed with weights in the Fourier plane (Equation (5)), followed by the amplitude replacement or modification with the measured diffraction data (Equation (6)):

(5) $$I_{j,i}={\left|\Psi_{j,i}\right|}^{2},\;I_{j,all}=\sum_{i=1}^{n}\eta_{j,i}I_{j,i}$$

(6) $$\Psi_{j,major}^{'}=\sqrt{I_{j,0}}\frac{\Psi_{j,major}}{\sqrt{I_{j,all}}},\;\psi_{j,major}^{'}=F^{-1}\left\{\Psi_{j,major}^{'}\right\}$$

where $\Psi_{j,i}=F\left\{\psi_{j,i}\right\}$ is the wave field of the *i*-th segment at the *j*-th scanning position, $I_{j,i}$ is the diffraction intensity of this segment, $I_{j,all}$ is the total diffraction intensity of all the segments at the *j*-th scanning position, $\eta_{j,i}$ is the time weight for the *i*-th segment at the *j*-th scan position, i.e., the ratio of the IVP number in this segment to the total number of IVPs of the *j*-th scan position, $I_{j,0}$ is the experimentally measured pattern for the *j*-th scan position, and $\Psi_{j,major}$ is the major segment diffracted wave of the *j*-th scan position, which is updated to $\Psi_{j,major}^{'}$. The updated exit wave of the major segment is $\psi_{j,major}^{'}$.

At this point, the major segment-related object function $O_{j,major}^{'}$ and the probe function $P^{\prime }$ are updated as follows:

(7) $$O_{j,major}^{'}=O_{j,major}+\alpha \frac{P^{*}}{{\left|P\right|}_{\max}^{2}}\left(\psi_{j,major}^{'}-\psi_{j,major}\right),\;P^{'}=P+\beta \frac{O_{j,major}^{*}}{{\left|O_{j,major}\right|}_{\max}^{2}}\left(\psi_{j,major}^{'}-\psi_{j,major}\right)$$

This method can effectively suppress the effect of random relative vibrations of the sample stage (with respect to the FZP) on the reconstructed image. In this study, the IVP distribution at each position was divided into six segments to eliminate the random vibration effects and to result in the accurately reconstructed probe, i.e., the accurate spot function we need.

Since the STXM imaging is performed by scanning the sample at the focal point of the FZP, the chosen PSF should also be the wavefront function of the focused spot. Typically, the ptychography reconstruction yields an out-of-focus spot that cannot be used directly as the PSF, but must be backpropagated to the focal point to obtain the correct focal spot function before being used as the PSF. The backpropagation equation is shown in Equations (8) and (9) [44]:

(8) $$I_{j}\left(r\right)=\;{\left|D_{z}\left\{\psi \left(r\right)\right\}\right|}^{2}$$

where $\psi \left(r\right)$ is the ptychographically reconstructed probe spot, a two-dimensional complex-valued function. Its intensity distribution after propagation is $I_{j}\left(r\right)$. $D_{z}$ is the propagator and denotes that the light wave propagates a distance z in free space. For near-field distances, $D_{z}$ is the angular spectrum propagator:

(9) $$D_{z}\left\{\psi \left(r\right)\right\}=\;F^{-1}\left[F\left[\psi \left(r\right)\right]\exp\{-2\pi iz/\lambda \sqrt{1-q^{2}\lambda^{2}}\}\right]$$

where λ is the wavelength, z is the propagation distance whose plus and minus signs have directional significance, F denotes the Fourier transform, and q is the reciprocal space coordinate.

In order to determine whether the probe backpropagated over a certain distance is the focal spot, we need a quantitative evaluation method. In this work, we used the sharpness value of the wavefront as the criterion to determine whether or not the probe has backpropagated to the focal point, where the sharpness will reach the maximum value. The sharpness of the backpropagated probe $S_{p}\left(r\right)$ is calculated as follows:

(10) $$S_{p}(z)={\sum_{x}\sum_{y}\left(D_{z}\left\{\psi \left(x,y\right)\right\}{D^{*}}_{z}\left\{\psi \left(x,y\right)\right\}\right)}^{2}$$

where $D_{z}\left\{\psi \left(x,y\right)\right\}$ is the wavefront function of the probe backpropagated over a distance z, and the right side of the equal sign is the two-dimensional area integration of the square of the intensity of the wavefront function. $S_{p}\left(z\right)$ is the sharpness value as a function of z, and when the sharpness function reaches its maximum value, we believe that the probe propagates to the focal point, and the resulting wavefront is the focal spot.

To investigate whether the ptychographically reconstructed focal spot function for a certain energy can be used in the deconvolution enhancement of the STXM images at other energies, i.e., whether there is a difference between the focal probe functions for different X-ray energies, we designed a simulation and experiment process to calculate or measure the focal spots at different energies and to compare their shapes and relative intensity distributions. The simulation results are attached in the Supplementary Material, while the experimental results are shown in Section 3.1 below.

Both simulation and experimental results show that the shape and relative intensity distribution of the focal spot remain essentially constant as the energy changes. Thus, in principle, the FZP focal spot obtained from the ptychography experiment at one energy can be used in the enhancement process of all STXM images at nearby energies without having to measure the probe once for each energy of the STXM imaging.

#### 2.2.2. Setup of Deconvolution Parameter

1. Intensity compression of reconstructed focal spot

Before applying the focal spot function to deconvolute the STXM image, we need to compress the overall intensity of the ptychography-based reconstructed focal spot. Although the intensity distribution of the focal spot obtained through ptychography reconstruction and backpropagation is consistent with that of the probe spot used in the STXM imaging, the imaging mechanisms of the two techniques are different: The mechanism of ptychography is light diffraction, and the data are acquired with an area detector; whereas the mechanism of STXM is light transmission and 2D integration of intensity, and the data are acquired with a point detector. The exposure time during the imaging process is also different between the two: it is generally 1 ms/pixel for STXM, whereas it is generally tens to hundreds of ms per position for ptychography. Therefore, the focal spot function obtained through ptychography cannot be directly used as the PSF for STXM deconvolution before compressing (dividing) the intensity in the overall by a coefficient *c*.

In this work, we first select a point in an STXM image where there is no absorption signal, i.e., there is no sample at that location, so that the transmittance of the light at that point is 1. Since the STXM image is the result of the convolution of the probe function with the sample function, when the focal spot scans over these points with a transmittance of 1, the intensity of the STXM image at that point is the result of the areal integral of the probe intensity function itself. Therefore, the compression coefficient c for the probe intensity is given by the following:

(11) $$c=\frac{i_{\max}\left(x,y\right)}{\sum_{x}\sum_{y}D_{focus}\left\{\psi \left(x,y\right)\right\}{D^{*}}_{focus}\left\{\psi \left(x,y\right)\right\}}.$$

where $i_{max}\left(x,y\right)$ is the maximum value of the STXM image intensity, $D_{focus}\left\{\psi \left(x,y\right)\right\}$ is the resulting wave function by the probe function $\psi \left(x,y\right)$ propagated to the focal point, and the 2D integral in the denominator gives the total power of the focal spot.

2. Noise-to-signal ratio (NSR)

According to Equations (3) and (4), if we want to obtain a better deconvolved image, the main parameter that needs to be optimized is the noise-to-signal ratio (NSR):

(12) $$NSR=\;\frac{S_{n}(f_{x},f_{y})}{S_{f}(f_{x},f_{y})}$$

In our practical processing, the NSR was set as a constant. The NSR value cannot be measured directly in experiments, and needs to be determined by optimization in the image enhancement processing. We took a series of NSR values in the range of 0.001~0.1 to try to perform the deconvolution calculations. By quantitatively analyzing the change of the deconvolved image quality (resolution) with the value of the NSR, we obtained the optimal value of the NSR and, at the same time, the optimal deconvolved image. For the same type of STXM images with close experiment times, such as the STXM images with similar X-ray energies and the same or similar sample types, their NSRs are actually the same or close to each other. Therefore, the optimal NSR obtained from the enhancement optimization of one of the STXM images is applicable to other STXM images of the same type without having to optimize it for each STXM image.

3. Evaluation of image quality

We adopt a quantitative criterion for evaluating the quality of a deconvolution image based on line profile gradient curves. In this evaluation method, the pixel value profiles of one or more lines that have a large contrast range and cross a certain boundary of the sample structure are taken from the image and differentiated to produce their gradient curves; the full widths at half maximum (FWHM) of these gradient curves are calculated (through Gaussian fitting) and used as the evaluation criterion for the quality of a deconvolved image. The smaller the FWHM of the gradient curve, the better the deconvolution result, and the FWHM can be directly regarded as the spatial resolution of the deconvolved image.

4. Pixel matching

Since the pixel size of the STXM images is determined by the scan step size, while the pixel size of the recovered focal spot by ptychography is jointly determined by the CCD pixel size, the CCD array size, the sample detector distance, and the X-ray energy, the pixel sizes of the two methods are probably not equal. As a result, the direct STXM image deconvolution using the ptychographic focal spot cannot be performed. Therefore, we need to perform pixel matching between the STXM image and the focal spot image. To achieve pixel matching, we use an upsampling operation on the two images, i.e., we upsample the two images to the same pixel size (which is smaller than the original pixel sizes of the two images). The upsampling is implemented through bilinear interpolation of the focal spot function (PSF) and the STXM image to obtain images with the same (and smaller) pixel sizes. Then, deconvolution calculations are performed according to the deconvolution principle described above (Equations (1)–(4)). This upsampling step does not increase the spatial resolution of the images, but ensures continuity and smoothing of intensity variation between pixels in the upsampled image. In this study, all probe functions and STXM images were interpolated to a 5 nm pixel size.

## 3. Results

### 3.1. Accurate Reconstruction of Focal Spot and STXM Image Enhancement with the Recovered Spot

**Figure 1:**
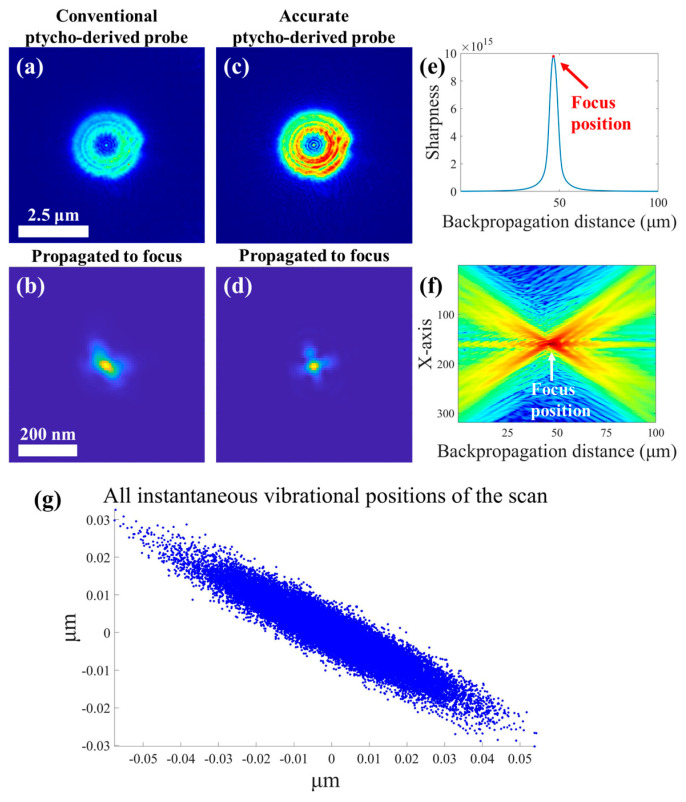
Acquisition of the focal spot at 710 eV. (**a**) The conventionally reconstructed probe by ptychography. (**b**) The focal spot obtained by backpropagation of the conventionally reconstructed probe. (**c**) The accurately reconstructed probe by the proposed strategy. (**d**) The focal spot obtained by backpropagation of the accurately reconstructed probe. (**e**) The variation curve of the probe spot sharpness with the backpropagation distance. The maximum of the curve corresponds to the focus position. (**f**) Cross-section of the light field on the x–z plane with backpropagation along the *z* axis. (**g**) The distribution of instantaneous vibrational positions in a ptychography scan.

The optimal FZP focal spot was obtained through the proposed accurate probe reconstruction strategy, and the verification experiment was carried out at the STXM endstation of the BL08U1A beamline of the Shanghai Synchrotron Radiation Facility (SSRF), Shanghai, China [45,46]. A Siemens star pattern was selected as the imaging sample and the incident X-ray photon energy was 710 eV. The FZP used had a diameter of 300 μm, an outermost zone width (OZW) of 30 nm, a central stop diameter of 80 μm, and an OSA of 70 μm. The sample was placed in an out-of-focus position to obtain an illuminating probe with a 2.5 μm diameter, so the ptychographically reconstructed probe must be backpropagated to the focus position to obtain the focal spot (we will call this focal spot the ptycho-derived focal spot in the following). As shown in Figure 1, the accurate reconstruction strategy for the probe eliminates the effects of vibration on the shape of the retrieved focal spot and yields an optimal focal spot function for subsequent image enhancement processing. The high-quality sample image was also simultaneously reconstructed by ptychography, which is placed in the Supplementary Material.

**Figure 2:**
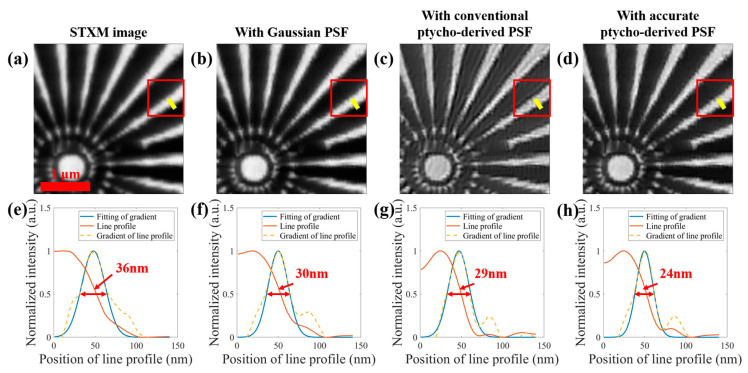
The Siemens star STXM image and its enhanced images: (**a**) STXM image; (**b**) the deconvolutionally enhanced image with a 2D Gaussian function of 36.6 nm width; (**c**) the enhanced image with the conventional ptycho-derived focal spot; (**d**) the enhanced image with the accurate ptycho-derived focal spot. (**e**–**h**) The results of line profile fitting to the averaged 22 lines (yellow lines and their parallels) in (**a**–**d**), respectively, showing the resolutions of the four images (in red).

We applied sequentially a 2D Gaussian function of 36.6 nm of the FWHM width, the conventional ptycho-derived focal spot, and the accurate ptycho-derived focal spot as the PSF to the STXM image deconvolution enhancement using the Wiener filtering algorithm, with the results shown in Figure 2. The spatial resolutions of the STXM image and the three enhanced images were derived by line profile fitting analysis at the same location of the four images, where the resolution of the STXM image was 36 nm, the resolution of the enhanced image with the Gaussian function was 30 nm, and the resolution with the conventional focal spot was 29 nm, while the resolution with the accurate focal spot was 24 nm. Compared with the STXM image, the image quality is improved after the deconvolution enhancement. Moreover, compared with the Gaussian function or the conventional ptycho-derived focal spot, the accurate ptycho-derived focal spot by the proposed strategy results in a significantly higher resolution of the enhanced image, indicating that the proposed accurate probe reconstruction strategy is effective and generates a more precise focal spot or PSF than the conventional reconstruction method for STXM deconvolution enhancement.

**Figure 3:**
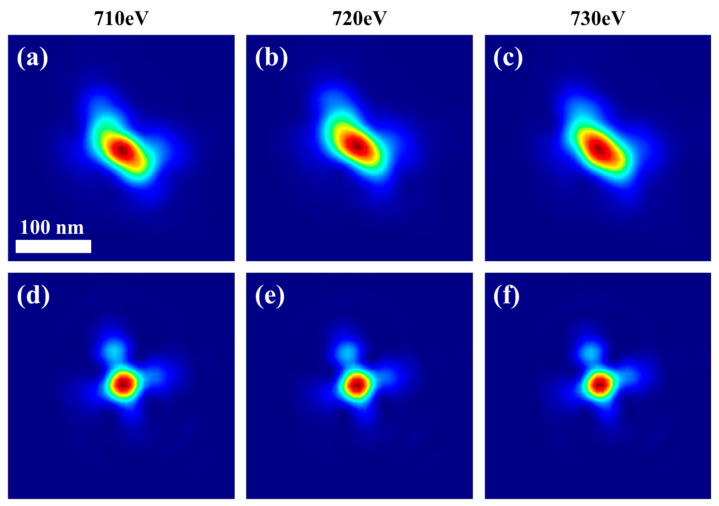
Focal spots based on ptychography reconstruction at energies of 710, 720, and 730 eV: (**a**–**c**) conventional ptycho-derived focal spot images at the three energies, respectively; (**d**–**f**) the accurate ptycho-derived focal spot images at the three energies, respectively.

In order to examine whether there is a difference between the focal spot functions of different X-ray energies, we selected three X-ray energies, 710 eV, 720 eV, and 730 eV, to perform ptychography imaging experiments on a Siemens star pattern to investigate whether the shape and intensity distribution of the reconstructed FZP focal spot vary with the X-ray energy. The experiments were carried out at the same beamline as the above experiment used [45,46]. The reconstructed out-of-focus probe diameter was 2.5 μm, and the focal spot images were obtained after optimizing the backpropagation distance. Figure 3 shows the conventional and accurate ptycho-derived focal spot images at the three energies, indicating that the accurate probe reconstruction strategy can eliminate random vibration effects on the retrieved focal spot shape, while giving very similar focal spots for the three energies. Meanwhile, for the accurate ptycho-derived focal spots at the three energies, we chose the normalized cross-correlation (NCC) [47] and the structural similarity index measure (SSIM) [48] as the basis for judging whether there are differences between the three focal spots. The NCC and SSIM are defined as follows:

(13) $$NCC\left(A,B\right)=\frac{\sum_{x}\sum_{y}\left(A\left(x,y\right)-\mu_{A}\right)\left(B\left(x,y\right)-\mu_{B}\right)}{\sqrt{\left(\sum_{x}\sum_{y}{\left(A\left(x,y\right)-\mu_{A}\right)}^{2}\right)\left(\sum_{x}\sum_{y}{\left(B\left(x,y\right)-\mu_{B}\right)}^{2}\right)}}$$

where A and B are the images, and $\mu_{A}$ and $\mu_{B}$ are the mean values of A and B, respectively.

(14) $$SSIM\left(A,B\right)=\frac{\left(2\mu_{A}\mu_{B}+0.01^{2}\right)\left(2\sigma_{AB}+0.03^{2}\right)}{\left({\mu_{A}}^{2}+{\mu_{B}}^{2}+0.01\right)\left({\sigma_{A}}^{2}+{\sigma_{B}}^{2}+0.03\right)}$$

where $\mu_{A}$ and $\mu_{B}$ are means of images A and B, respectively, $\sigma_{A}$ and $\sigma_{B}$ are the variances of A and B, respectively, and $\sigma_{AB}$ is the covariance between A and B.

The comparison process of the accurate focal spot functions at two energies of E1 and E2 is labeled as $F\left(p_{E1},p_{E2}\right)$, and the comparison results for the three accurate focal spots are shown in Table 1, which indicates that the shape and relative intensity distribution of the accurately reconstructed focal spot by the proposed strategy remains basically constant as the photon energy changes.

Thus, in theory, the FZP focal spot obtained from the ptychography experiment at one energy can be used in the enhancement process of all the STXM images at nearby energies without having to measure the probe once for each energy of the STXM imaging. This provides a constant focal spot base for the tractability of the subsequent STXM image enhancement.

By optimizing and analyzing the focus position, energy relevance, and stability of the X-ray probe, a high-quality ptychography-based accurate focal spot function can be obtained, which will be applicable in subsequent deconvolution enhancement processing of a series of STXM images. Based on this accurate focal spot and the developed image deconvolution enhancement approach, we can obtain the optimal inverse filter function and other deconvolution parameters to obtain the enhanced STXM images of the best quality.

### 3.2. STXM Image Enhancement with Different Inverse Filters Based on Accurately-Recovered Focal Spot

**Figure 4:**
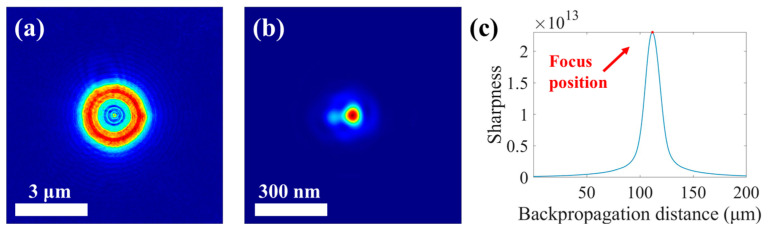
Acquisition of the focal spot at 778 eV in the experiment for Pt-Co nanoparticles. (**a**) The probe reconstructed by ptychography, which has a diameter of 3 μm. (**b**) The focal spot obtained by backpropagation of the reconstructed probe. (**c**) The variation curve of the probe spot sharpness with the backpropagation distance. There exists the maximum value in the curve, and its corresponding light spot is the focal spot.

To further verify the effectiveness of the developed STXM image enhancement technique based on the ptychography-recovered focal spot, the following experiment and data processing were performed in this study. In the experiment, Pt-Co alloy nanoparticles were used as the sample for the STXM imaging with a scanning step of 50 nm and an X-ray energy of 776.6 eV. The FZP used in the experiment had a diameter of 240 μm, an OZW of 60 nm, and a central stop with a 100 μm diameter. The focal spot was derived from the reconstructed probe by ptychography at 778 eV energy. The reconstructed probe size was 3 μm, and the focal spot was obtained after backpropagation to focus. The variation curve of the backpropagated probe spot sharpness with the backpropagation distance and the probe spots before and after propagation are shown in Figure 4.

**Figure 5:**
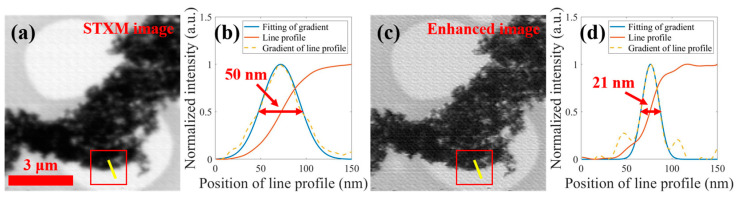
The enhancement of the STXM image of Pt-Co nanoparticles using the Wiener filter algorithm: (**a**,**c**) show the STXM image and its enhanced image, respectively; (**b**,**d**) show the results of line profile fitting to the 189 lines (yellow lines and their parallels, averaged) in (**a**,**c**), respectively, indicating that the resolution of the STXM image is 50 nm while the resolution of the enhanced image is 21 nm.

After obtaining the focal spot function, we applied it as a PSF in the deconvolutional enhancement processing of the STXM image from this experiment using the Wiener filter algorithm. The optimal enhanced image was obtained by compressing the spot intensity and optimizing the NSR parameter to 0.007; the results are shown in Figure 5. By comparing the STXM image with the enhanced image, it can be seen that many local details that are not clearly visible in the STXM image are clearly displayed in the enhanced image, and the overall “haze” of the image has been reduced with the clarity being significantly improved. The spatial resolutions of the two images were derived by line profile fitting analysis at the same location of the two images, and the resolution of the STXM image was 50 nm, while the resolution of the enhanced image was 21 nm, indicating that the quality of the enhanced image was significantly better than that of the STXM image. This demonstrates that the developed image enhancement approach is effective for optimizing the quality of the STXM images.

**Figure 6:**
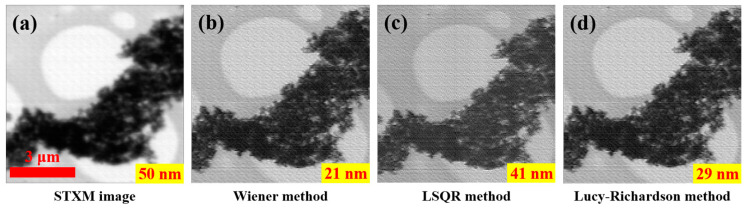
The enhancement of the STXM image of Pt-Co nanoparticles using three deconvolution methods: (**a**) the STXM image; (**b**) the enhanced image using the Wiener filtering method; (**c**) the enhanced image using the LSQR method; (**d**) the enhanced image using the Lucy–Richardson method. The resolution of each image is labeled at the lower-right corner of each image.

In addition to the image enhancement processing using the Wiener inverse filter, we also used other inverse filtering methods, such as the LSQR method and the Lucy–Richardson deconvolution method, to perform image enhancement processing, as shown in Figure 6. The line profile analysis results show that the resolutions by these methods are all better than that of the STXM image, indicating that other deconvolution methods are also able to improve the STXM image quality based on the accurate ptychographic probe. As different methods have different sensitivities to noise, they also differ in the image resolution after the enhancement processing.

Figure 6 shows that the resolution of the enhanced image using the Wiener filtering method is the highest, followed by the enhanced images using the Lucy–Richardson method and the LSQR method. The Wiener filtering method has the most stable image enhancement results, while the Lucy–Richardson method is very efficient when the PSF is known. The LSQR method has a high memory requirement and is more sensitive to the noise in the raw image, so that its deconvolution results are not stable.

### 3.3. Enhancing STXM Images with Different Scan Step Sizes

In STXM imaging, the resolution is determined by the scan step size when the scan step size is greater than the focal spot size, but is approximately equal to the focal spot size when the step size is less than or equal to the focal spot size. It is reasonable to assume that the enhanced processed STXM images with different step sizes will also differ in resolution, and it is possible that the resolution of the enhanced image will surpass the spot size limitation and reach a level smaller than that of the focal spot when the scan step size is smaller than the focal spot. To demonstrate this, we conducted several STXM imaging experiments and simulations in which the scan step size varied around the spot size, to observe how our image enhancement approach improves resolution beyond the focal spot size limit. The steps and results of the simulations are in the Supplementary Material, while the experimental steps and results are presented below.

**Figure 7:**
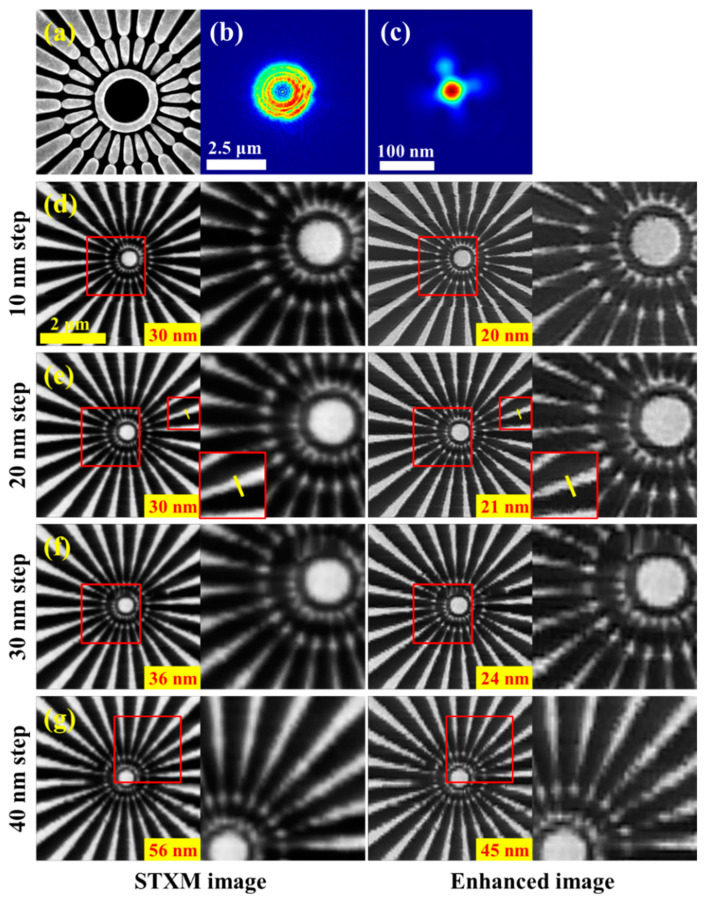
Experimental STXM images with scan steps from 10 to 40 nm and their enhancement processed images, the imaging sample is a Siemens star pattern. (**a**) The SEM image of the sample. (**b**) The accurate probe reconstructed by ptychography, which has a diameter of 2.5 μm. (**c**) The accurate focal spot obtained by backpropagation of the probe in (**b**). The focal spot size is 30 nm. (**d**–**g**) Show the STXM images and their enhanced images with step sizes of 10 nm, 20 nm, 30 nm, and 40 nm, respectively. In each row of panels, the first panel is the STXM image, the second is a zoomed-in view of the red-boxed area in the first panel, the third is the enhanced image of the first panel using the developed strategy, and the fourth is a zoomed-in view of the red-boxed area in the third panel. The resolution obtained through line profile analysis is indicated in the lower right corner of each panel in the first and third columns of (**d**–**g**).

The STXM experiments with four different scan steps and a ptychography experiment were performed at the STXM endstation of the SSRF. In the experiment, a Siemens star pattern was selected as the imaging sample (Figure 7a), the incident X-ray energy was 710 eV, and the probe spot was generated from a 300-μm-diameter FZP with an OZW of 30 nm, thus the focal spot size was about 30 nm. Four scanning steps of 10 nm, 20 nm, 30 nm, and 40 nm were used for the STXM imaging. The ptychography-reconstructed accurate probe and the recovered focal spot by backpropagation are shown in Figure 7b and Figure 7c (intensity distribution plots), respectively. Figure 7d–g show the STXM images and their enhancement processed images using the Wiener filter algorithm for the four scan steps.

**Figure 8:**
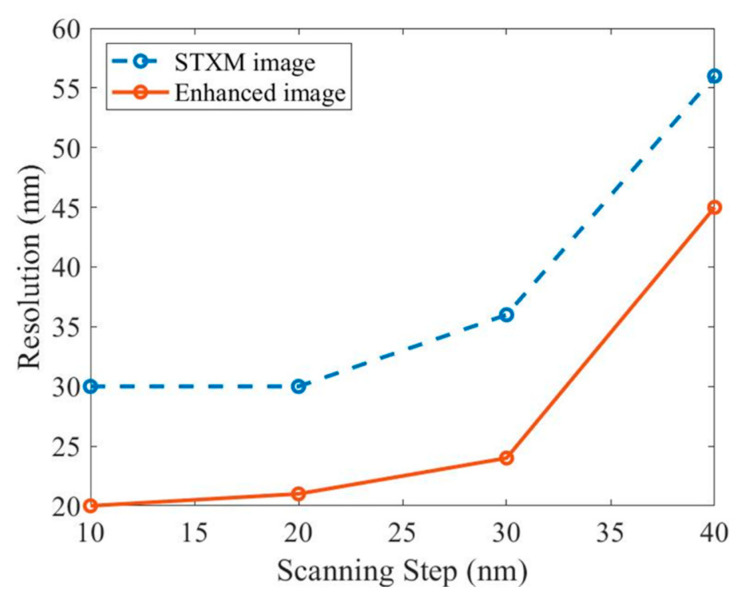
Resolution variations of experimental STXM images and their enhanced images with the scan step size. The resolutions of the enhanced images are significantly higher than that of the STXM images for the four scan step sizes, even breaking through the focal spot size (30 nm) limitation when the scan step is <40 nm.

By comparing the STXM images with their enhancement processed images for different scanning steps, it can be seen that the developed image enhancement approach can effectively improve the quality of the STXM images with various scanning steps, and the resolution of the enhanced images increases steadily with the reduction of the scan step size. Through the line-profile fitting method, we obtained the resolution values of all the STXM images and their enhanced images, as labeled in each image of Figure 7 and displayed in Figure 8. The results show that, as the scan step increases from 10 to 40 nm, the resolution of the STXM image is, in order, 30 nm, 30 nm, 36 nm, and 56 nm, while the resolution of the corresponding enhanced image is 20 nm, 21 nm, 24 nm, and 45 nm, respectively. It is evident that the developed image enhancement approach improves the quality of the STXM images of different scan step sizes using the same focal spot from ptychography. Moreover, when the scan step is less than 40 nm, the enhanced image resolution breaks through the STXM limit, i.e., the focal spot size, and achieves sub-focus resolution.

### 3.4. Enhancing STXM Images at Different Energies (Stack Data)

**Figure 9:**
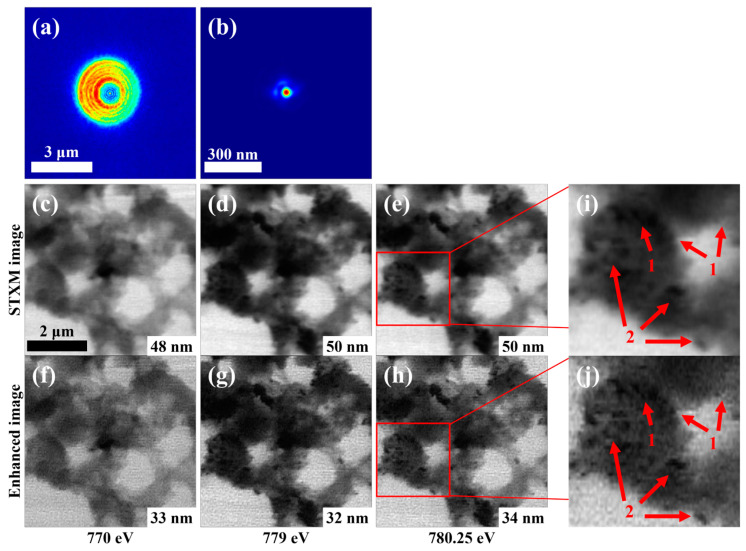
Three experimental STXM images at different energies and their enhancement processing results. The sample is NiCoP electrode particles: (**a**) the ptychography-reconstructed probe image at 770 eV; (**b**) the focal spot obtained by backpropagation of the probe in (**a**). (**c**–**e**) Show the STXM images at energies of 770 eV, 779 eV, and 780.25 eV, respectively. (**f**–**h**) Show the enhanced images of (**c**–**e**), respectively. The resolution of each image is labeled in the lower right corner of each panel of (**c**–**h**). The red-boxed areas in (**e**,**h**) are amplified in (**i**,**j**), respectively, showing that the structures and shapes of local substances are clearer in the enhanced image, providing new information about the particles.

To investigate whether the ptychographically recovered probe function at one energy can be applied to the deconvolutional enhancement processing of the STXM images at other different energies, we performed image enhancement processing and analysis on a set of energy-stacked STXM data from an experiment performed at the BL08U1A beamline of the SSRF. In the experiment, NiCoP electrode material particles were used as a sample with X-ray energy ranging from 770 eV to 800 eV and a STXM scan step of 25 nm. The focal spot function used in the enhancement process was derived from a ptychography-reconstructed probe function (2.5 μm in diameter) at an energy of 770 eV, which was backpropagated to obtain the focal spot of 40 nm in size. The X-ray probe images before and after backpropagation are shown in Figure 9a,b, respectively. The STXM images at three energies, 770 eV, 779 eV, and 780.25 eV, are shown in Figure 9c–e, and their enhanced images are shown in Figure 9f–h, respectively.

The resolution of each image was obtained by line profile gradient fitting and labelled in the lower right corner of each image (Figure 9c–h). The results show that the resolution of the STXM image at 770 eV is 48 nm and the resolution of its enhanced image is 33 nm, while the corresponding two resolutions at 779 eV are 50 nm and 32 nm, respectively; the corresponding two resolutions at 780.25 eV are 50 nm and 34 nm, respectively. The resolutions of the enhanced images at all three energies are significantly improved over those of the STXM images. Therefore, the developed image enhancement approach can be applied to process multi-energy stacked images, i.e., the resolutions of the whole series of energy stacked images can be enhanced by using the accurate focal spot from a single energy ptychography, indicating that this approach has a high applicability to different STXM imaging modes.

These results also show that the developed approach not only leads to an increase in sharpness, but reveals new details of the sample structure after deconvolution. From Figure 9 we can see that the unclear structures and blurred shapes of particle-like substances in the STXM images become clearer and well-defined after deconvolution, as denoted by the arrows in Figure 9i,j, where the substances (arrows group 1) are indeterminate as to whether they are particles in the STXM image, but are determined to be multiple particles in the enhanced image; meanwhile, the shapes of the particles (arrows group 2) are blurred in the STXM image, but are well-defined in the enhanced image. These comparisons suggest that new structural information or new spatial frequencies are provided by the enhanced images.

## 4. Discussion

The accuracy of the focal spot function or the PSF is a key factor to obtain a high-quality deconvolved STXM image. In 1991, Jacobsen et al. [27] proposed an indirect measure method for the PSF by combining the STXM and STEM images, which produced an approximate focal spot for STXM. In 2018, Ornelas et al. [29] utilized a model probe as the PSF to deconvolve the STXM images. In 2023, Abe et al. [35] incorporated probe corrections in the deconvolution process. In our study, we developed a precise ptychographic probe reconstruction strategy capable of separating the effects of vibrations from the probe reconstruction results, thereby obtaining an accurate probe and an accurate focal spot. Meanwhile, we demonstrated theoretically and experimentally that the PSF of an FZP is almost the same for an X-ray energy range around an elemental absorption edge. Therefore, the PSF reconstructed from a small-area ptychography imaging at one energy, can be used to deconvolve a whole series of STXM images at near energies and near times.

The achievable resolution by the developed image enhancement strategy, as indicated by the above results, can reach a level of less than the OZW of the FZP used when the scan step size is close to or less than the OZW. The resolution was evaluated based on the line profile gradient curve fitting in this work. The data of realistic samples, such as the Pt-Co nanoparticles and NiCoP electrode material, were used to test our deconvolution strategy. The resolution of the enhanced image of the Pt-Co sample exceeds the OZW, while the resolution of the NiCoP sample is close to, but not better than, the OZW, The resolution difference between the two cases is probably due to the different noise levels of the two experiments.

The data processing time required by this approach is much shorter than that of ptychography. Ptychography data processing or reconstruction typically takes several to tens of minutes due to its computational complexity, and in many cases, parameter optimization and additional processes to deal with instability, such as scan positions, sample–detector distance, and beam vibration, need to be performed iteratively, which exponentially increases the time spent and requires a high level of computational power. On the other hand, the image deconvolution enhancement typically takes less than a minute, and the parameters involved can be generalized across the different STXM images in this experiment. In general, the image enhancement computation requires very little computational power.

## 5. Conclusions

In this work, we developed and validated a ptychography-assisted STXM enhancement imaging approach. For the first step of this approach, we designed an accurate reconstruction strategy for a probe spot based on ptychography, which eliminates vibration effects on the retrieved focal spot shape. The accurately reconstructed probe function is backpropagated to the focal point and its intensity is compressed overall. Then, the resulting focal spot is used to enhance the STXM images with a deconvolution algorithm, thus obtaining a higher resolution image. For example, the enhanced STXM image of a Pt-Co nanoparticle sample shows 1.38 times increase in resolution from 50 nm to 21 nm. The approach can also process all STXM images of an energy stack using the ptychographically reconstructed probe function at one energy. Based on the stability consideration for the incident X-ray beam, the focal spot from the ptychography experiment in the same day or recent days should be selected as the point spread function for enhancement processing of the STXM images in practice.

We also systematically investigated the influence of the scan step size on the quality of the enhancement-processed images. Both simulations and experiments show that the developed image enhancement processing approach significantly improves the resolution of the STXM images with different step sizes, and the resolution of the enhanced images is further improved with the reduction of the scan step size; when the scan step size is varied in the vicinity of the focal spot size, the resolution of the enhanced image is able to break through the focal spot size limitation and reach the resolution of the sub-focus level. To some extent, this image enhancement approach alleviates the STXM resolution bottleneck caused by focusing elements.

## Figures and Tables

**Table 1:** NCCs and SSIMs between the accurate ptycho-derived focal spots at three energies. The results show that the shape and relative intensity distribution of the accurate focal spot remain essentially constant as the energy changes.

Focal Spots Comparison	SSIM	NCC
$F\left(p_{710\mathrm{eV}},p_{720\mathrm{eV}}\right)$	0.9621	0.9594
$F\left(p_{720\mathrm{eV}},p_{730\mathrm{eV}}\right)$	0.9820	0.9975
$F\left(p_{710\mathrm{eV}},p_{730\mathrm{eV}}\right)$	0.9697	0.9626

## Data Availability

Data will be made available upon reasonable request.

## Supplementary Materials

The following supporting information can be downloaded at: https://www.mdpi.com/article/10.3390/nano15070496/s1, Figure S1: Simulation of focal spots at different energies; Figure S2: Ptychography-reconstructed Siemens star and probe images with the designed accurate reconstruction strategy; Figures S3 and S4: Simulation of STXM image enhancements for different scan step sizes. Ref. [49] is cited in the supplementary materials.
