# Supplementary material for: Enhanced Imaging in Scanning Transmission X-Ray Microscopy Assisted by Ptychography
Source: Nanomaterials (Basel). 2025 Mar 26;15(7):496. doi: 10.3390/nano15070496 (PMC11990249; doi:10.3390/nano15070496)
Supplement: Supplementary file 1 [file nanomaterials-15-00496-s001.zip › nanomaterials-3500030-supplementary.pdf]

Simulation of focal spots at different energies

In the simulation, the FZP diffraction integral formula [1] was used to generate the focal spot wavefront functions for X-ray energies of 700~703 eV, 730 eV and 900 eV, respectively (Figures S1(a-f)). The parameters of the FZP used were: diameter 300 μm, width of the outermost zone 30 nm, diameter of the central stop 80 μm, and aperture of the OSA 70 μm. The focal spot images of different energies were adjusted (by bilinear interpolation) to the same pixel size (5 nm), and a line profile was taken horizontally across the center of the spot for each energy (Figure S1(g)). The shapes and relative intensities of all orders of diffraction rings in these line profiles were compared, showing that the shape and intensity distribution of the FZP focal spot remain unchanged as the energy changes.

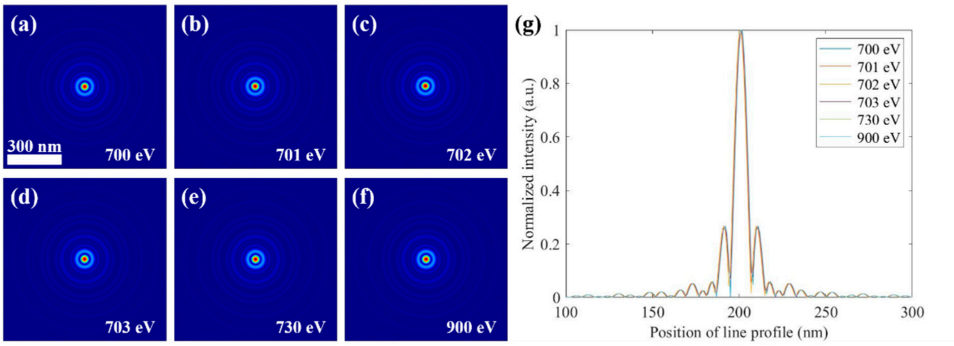

Figure S1. Focal spots obtained from the FZP theoretical model at 700~900 eV photon energies. (**a**-**f**) The intensity images of the focal spots at energies of 700 eV~703 eV, 730 eV, and 900 eV, respectively, with the same pixel size of 5 nm. (**g**) the line profiles across the center of each spot shown in (**a**-**f**). (**g**) shows that the shapes and intensity distributions of the spots are almost identical at these energies.

Ptychography-reconstructed Siemens star and probe images with the developed accurate reconstruction strategy

To obtain the optimal FZP focal spots by the accurate probe reconstruction strategy we developed, a ptychography experiment for a Siemens star imaging was carried out at the STXM endstation of the BL08U1A beamline of the Shanghai Synchrotron Radiation Facility, as described in Section 3.1 of the paper. In addition to the accurate probe function, a high-quality sample image was also simultaneously reconstructed by ptychography, as shown in Figure S2.

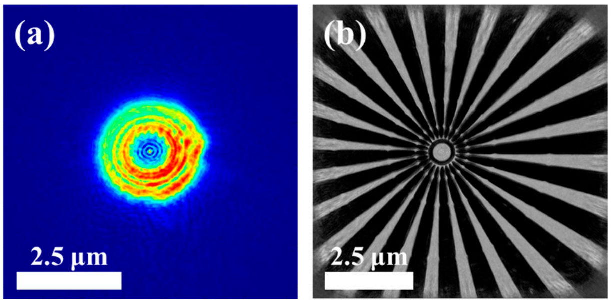

Figure S2. Reconstructed probe image and object image at 710 eV from a Siemens star ptychography data. (**a**) the reconstructed probe image. (**b**) the reconstructed sample image.

Simulation of STXM image enhancements for different scan step sizes

The simulation assumed that both the sample and probe images had a pixel size of 10 nm, and the probe spot (FZP focal spot) had a size of 30 nm. The focal spot was generated in the same way as shown in Figure S1. Figures S3(a-c) show the original sample image, the focal spot image, and the logarithmic focal spot image, respectively. The STXM image was obtained by convolving the sample function with the focal spot function at each scanning point and adding random noise. Scanning was performed using five step sizes of 10 nm, 20 nm, 30 nm, 40 nm, and 50 nm. The corresponding STXM images and enhancement processing results are shown in Figures S3(d-h), respectively. Figures S3(i-j) illustrate the image resolution calculation process for an STXM image and its enhanced image by the line-profile gradient curve fitting.

Observing the five sets of images in Figures S3(d-h), it can be seen that the resolution of the simulated STXM images (in the left two columns) slightly improves with the reduction of the scan step size, but remains unchanged once the step size is less than 30 nm. On the other hand, the resolution of the enhanced image steadily improves with the reduction of the scan step size, and is not limited by the focal spot size. Comparing the STXM images and the enhanced images for different scan steps, it can be seen that the developed image enhancement approach significantly improves the quality of STXM images at various step sizes. By the line-profile fitting method shown in Figures S3(i-j), we obtained the specific resolution values of all the simulated STXM images and their enhanced images, as labeled in Figure S3 and displayed in Figure S4. The results show that as the scan step increases from 10 to 50 nm, the resolution of the STXM image changes from 30 nm to 50 nm, while the resolution of the corresponding enhanced image changes from 12 nm to 35 nm. It is evident that the developed image enhancement approach significantly enhances the resolution of STXM images at all five scan step sizes using the same focal spot. Moreover, the enhanced image resolution can break through the focal spot size (30 nm) limitation on the STXM resolution when the scan step is ≤ 40 nm.

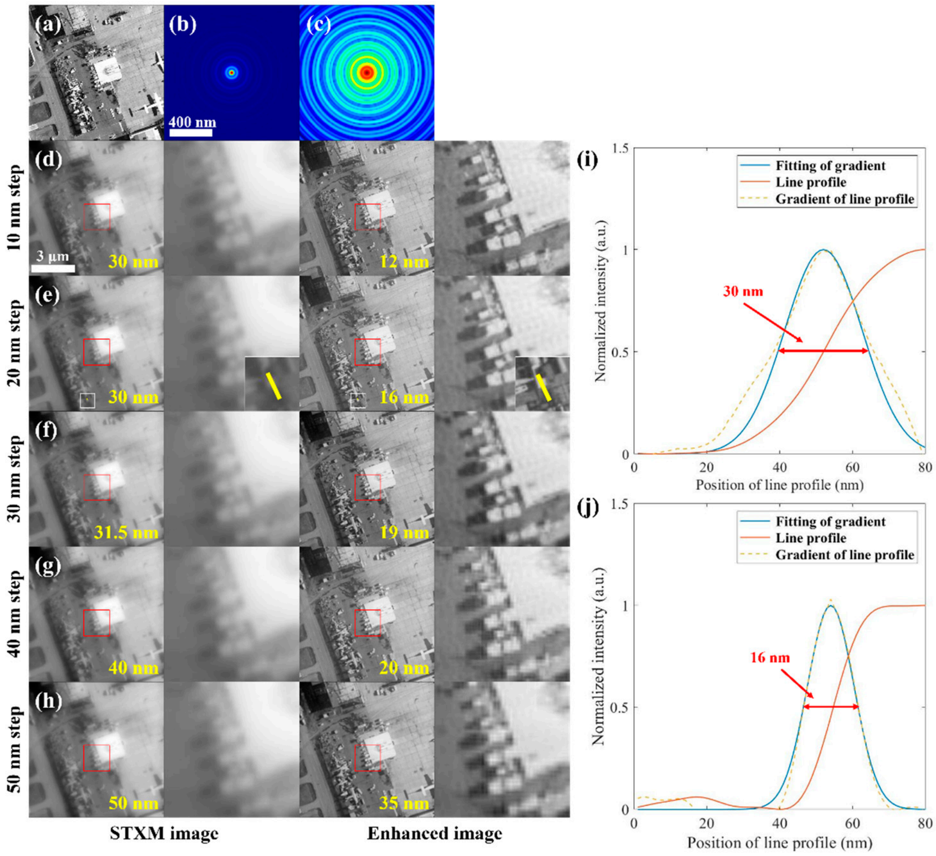

Figure S3. Simulated STXM images with scan step sizes from 10 to 50 nm and their enhancement processed images. (**a**) the sample image used in the simulation. (**b**) the focal spot image. (**c**) the logarithmically plotted focal spot image. The spot size (FWHM) is 30 nm. (**d**-**h**) show the STXM images and their enhanced images with step sizes of 10 nm, 20 nm, 30 nm, 40 nm, and 50 nm, respectively. In each row of panels, the first panel is the STXM image with the corresponding step size, the second panel shows an enlarged red-boxed area in the first panel, the third panel is the enhanced image of the first panel by using the developed approach, and the fourth panel shows an enlarged red-boxed area in the third panel. The yellow lines (labeled in (**e**)) in the 20-nm-step STXM image and its enhanced image are used for line-profile gradient curve fitting to obtain the resolution, and the results (30 nm and 16 nm) are shown in (**i**-**j**), respectively. The resolution calculated in this way is indicated in the lower right corner (yellow number) of each panel in the first and third columns of Figures (**d**-**h**).

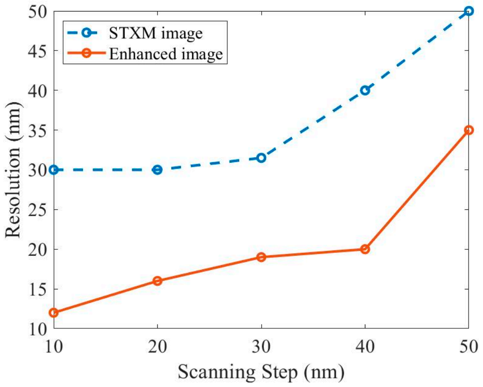

Figure S4. Resolution variations of simulated STXM images and their enhanced images with the scan step size (10~50 nm) by the developed approach. The resolutions of the enhanced images are significantly higher than that of the STXM images for the five scan step sizes, even breaking through the focal spot size (30 nm) limitation when the scan step is ≤ 40 nm.

Reference

1. Attwood, D. *Soft X-Rays and Extreme Ultraviolet Radiation: Principles and Applications*; Cambridge university press, 2000.

Disclaimer/Publisher’s Note: The statements, opinions and data contained in all publications are solely those of the individual author(s) and contributor(s) and not of MDPI and/or the editor(s). MDPI and/or the editor(s) disclaim responsibility for any injury to people or property resulting from any ideas, methods, instructions or products referred to in the content.
